# Case Report: A case of thoracoscopic mediastinal tumor resection in a child with ROHHAD syndrome

**DOI:** 10.3389/fped.2024.1450017

**Published:** 2024-09-13

**Authors:** Yangwei Ma, Jia Gao, Lianghong Huo, Fang Wang

**Affiliations:** Department of Anesthesiology, Beijing Children’s Hospital, Capital Medical University, National Center for Children’s Health, Beijing, China

**Keywords:** ROHHAD syndrome, mediastinal tumor, neuroblastoma, hypernatremia, anesthetic management, case report

## Abstract

Rapid-onset obesity with hypoventilation, hypothalamic dysfunction, and autonomic dysregulation (ROHHAD) is an exceptionally rare condition. This case report highlights a child diagnosed with ROHHAD syndrome, presenting with a mediastinal tumor. ROHHAD syndrome is characterized by early onset obesity, hypothalamic dysfunction, autonomic dysfunction, inadequate ventilation, suspected seizures, and abnormal behavior. The presence of a mediastinal tumor necessitated surgical intervention. Key considerations during surgery included hypernatremia due to hypothalamic dysfunction, potential airway challenges, preoperative anemia, and hemodynamic fluctuations during the removal of the sizable mediastinal tumor. Comprehensive preparations ensured a safe operation. Notably, some children with this syndrome may exhibit symptoms such as decreased gastrointestinal function, polyuria, and thermoregulatory disturbances. Vigilance is essential during anesthesia assessment in these patients. Anesthesiologists should enhance their knowledge of this condition and tailor their management strategies based on individual clinical presentations and the specific planned surgical procedures.

## Introduction

Rapid-onset obesity with hypoventilation, hypothalamic dysfunction, and autonomic dysregulation (ROHHAD) syndrome is extremely rare, with fewer than 200 cases reported worldwide (1–3). It has been reported that 40%–56% of affected children have neuroendocrine tumors (1, 2, 4).

## Case description

A 4-year-old girl, standing 102 cm tall and weighing 26 kg, with a body mass index (BMI) of 24.99, was diagnosed with neuroblastoma more than 3 months ago. She is now preparing for thoracoscopic mediastinal tumor resection surgery.

Over a year before her hospital admission, the patient began to show an unexplained increase in weight while maintaining a normal appetite and consuming minimal water. Additionally, she exhibited abnormal behaviors and faced intermittent challenges with falling asleep at night, with suspected seizure episodes also being reported. Five months prior to admission, she developed strabismus and ptosis, with no discernible cause. While the initial diagnosis indicated severe astigmatism and myopia, no treatment was administered. Four months prior to admission, the patient exhibited generalized edema, particularly in the limbs and abdomen, along with respiratory distress, lip cyanosis, and significantly impaired mental acuity. The patient underwent a chest computed tomography (CT) scan at a local hospital, which revealed pneumonia with partial collapse of the right lower lobe and bilateral pleural effusion. Furthermore, an occupying lesion was discovered in the left upper mediastinum in addition to pericardial effusion. Following admission to the intensive care unit (ICU), the patient showed improvement after receiving tracheal intubation and mechanical ventilation support, along with the administration of albumin and antibiotics for infection control. Finally, more than 3 months before admission, the patient was transferred to our emergency department, where an ultrasound-guided biopsy of the mediastinal mass suggested the presence of a ganglioneuroblastoma.

Upon admission, a CT scan of the chest disclosed a mediastinal mass measuring 7.6 × 6.2 × 7.1 cm, along with left clavicular lymph node metastasis. Comprehensive chromosomal and whole exome sequencing revealed no genetic anomalies. Biochemical assessments revealed hypernatremia (147–162 mmol/L) and hyperchloremia (113–126 mmol/L). Moreover, a hormonal evaluation revealed hyperprolactinemia (149.18 ng/ml), the normal range is 3.1–15.7 ng/ml. Following four cycles of chemotherapy (CBVP [Carboplatin and Etoposide] twice, followed by CADO [Cyclophosphamide, Vincristine, and Pirarubicin] twice), a detailed evaluation of hypothalamic function was undertaken, revealing a significant increase in serum sodium levels (161.1 mmol/L). The implementation of enhanced fluid intake and a low-osmolar potassium-containing solution resulted in a modest reduction in blood sodium levels (152.1 mmol/L). However, prolactin levels remained markedly elevated at 110.04 ng/ml, alongside normal urine osmolality and cortisol rhythm. Ophthalmological examination disclosed partial visual field defects. Considering the patient's early-onset obesity, hypothalamic dysfunction manifesting as hypernatremia and hyperprolactinemia, and autonomic disturbances including excessive sweating, strabismus, ptosis, and sleep disturbances, coupled with inadequate ventilation (percutaneous oxygen saturation fluctuating between 85% and 90% without supplementary oxygen during sleep), and indications of seizures and aberrant behavior, a diagnosis of ROHHAD syndrome was established, along with a suspected mediastinal ganglioneuroblastoma.

Based on the information provided, the patient's medical, personal, and family histories are unremarkable. Preoperative evaluation revealed that the patient was alert, responsive, and active, with stable breathing and an oxygen saturation of 95%–98% without supplemental oxygen. The extent of mouth opening was adequate, but her cooperation during the physical examination was poor. Examination findings included left eye exotropia, ptosis, and slightly cool hands, with no other significant abnormalities.

**Figure 1:**
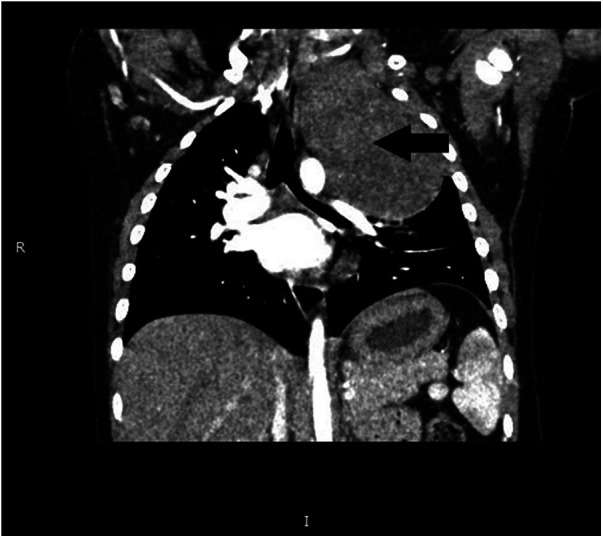
Chest CT results of the patient. Arrow: mediastinal tumor.

The preoperative assessment revealed a hemoglobin level of 10.2 g/dl, sodium concentration of 158.3 mmol/L, and chloride levels of 122.4 mmol/L. A chest CT scan (Figure 1) depicted a substantial, enhancing mass on the left side of the spine, measuring approximately 7.3 × 5.9 × 8.6 cm. This mass induced compression of the airway without causing significant stenosis, and patchy infiltrates were observed in the left lower lobe. Echocardiography revealed a patent foramen ovale measuring 1.9 mm with preserved left ventricular function. All other laboratory findings were within the normal ranges. The diagnosis was suggested to be a mediastinal ganglioneuroblastoma.

The child fasted routinely before surgery and received an intravenous infusion of a glucose-sodium chloride-potassium injection solution consisting of 8% glucose, 0.18% sodium chloride, and 0.15% potassium chloride for fluid therapy. Upon entering the operating room, standard monitoring was conducted, including measurements for SpO_2_, electrocardiogram, and non-invasive blood pressure. Subsequently, the child was administered 20 μg of fentanyl, 40 mg of propofol, and 2.5 mg of cisatracurium. Tracheal intubation was performed once the desired depth of anesthesia was achieved. A fiber bronchoscope guided the placement of a number 5 bronchial blocker into the left main bronchus, initiating single-lung mechanical ventilation in volume ventilation mode. The settings included a 75% oxygen concentration, an expiratory tidal volume of 200 ml, and a respiratory frequency of 22/min. Radial artery and femoral vein cannulation (5.0 double lumen) were conducted under ultrasound guidance, accompanied by bispectral index and temperature monitoring. Additionally, a urinary catheter was inserted.

During the surgery, a combination of sevoflurane (1%), propofol (6–10 mg/kg/h), and remifentanil (0.2–0.3 μg/kg/min) was administered to maintain anesthesia. The anesthetic drug dosages were adjusted according to the patient's bispectral index score, heart rate, and arterial blood pressure. Arterial blood gas levels were monitored throughout surgery, and the results are presented in Table 1.

**Table 1:** Arterial blood gas analysis results at different time points during the surgery.

	After arterial puncture	After the surgery begins	After tumor resection	After thoracic lavage
pH	7.318	7.315	7.367	7.292
PCO_2_ (mmHg)	46	46.5	40.1	47.7
PO_2_ (mmHg)	307	246	323	171
Beecf (mmol/L)	−3	−3	−2	−4
HCO_3_-(mmol/L)	23.6	23.7	23.1	22.9
Na^+^ (mmol/L)	155	154	154	149
K^+^ (mmol/L)	3.8	3.9	4.1	4.1
iCa (mmol/L)	1.37	1.38	1.19	1.34
Glu (mmol/L)	18.5	11.4	7.9	14.3
Hb (g/dl)	10.2	10.9	10.5	9.9

Beecf, base excess of extracellular fluid; Glu, glucose; Hb, hemoglobin; HCO_3_-, bicarbonate ion; iCa, ionized calcium; K+, potassium ion; Na+, sodium ion; PCO_2_, partial pressure of carbon dioxide; PO_2_, partial pressure of oxygen.

During surgery, a solid tumor with a rich vascular network was observed in the posterior mediastinum at the level of the C7-T6 vertebrae. The tumor surrounded the left subclavian artery, the vertebral artery, and multiple branches, with the brachial plexus nerves traversing through it. The surgical team meticulously dissected and protected the vessels and nerves, successfully completing tumor resection within a 3 h timeframe.

The patient was administered the following intravenous fluids postoperatively: 400 ml of glucose-sodium chloride-potassium solution, 100 ml of compound sodium lactate solution with mannitol, 400 ml of 5% glucose solution (500 ml + 6 units of insulin + 0.6 g of KCl), 1 unit of suspended red blood cells, and 200 ml of fresh frozen plasma. The patient experienced a blood loss of 10 ml and a urine output of 50 ml. Subsequently, the patient was transferred to the ICU with a single-lumen endotracheal tube.

Upon transfer to the ICU, the patient received mechanical ventilation, and an arterial blood gas analysis was promptly performed, as detailed in Table 2. One hour after ICU admission, the endotracheal tube was withdrawn, and nasal cannula oxygen therapy was administered at a flow rate of 2 L/min. Subsequent blood gas analysis was performed after another hour (Table 2), with routine arterial blood gas monitoring thereafter (Table 2). On the second postoperative day, the patient was transferred to the thoracic surgery ward and discharged on the fifth postoperative day. We recommended that this patient receive noninvasive positive pressure ventilation during sleep after discharge. Histopathological examination confirmed the diagnosis of neuroblastoma with ganglioneuroblastic differentiation.

**Table 2:** Results of arterial blood gas analysis at different time points after admission to the ICU.

	After transfer to the ICU	Removal of endotracheal tube	Postoperative day 1	Postoperative day 2
pH	7.639	7.319	7.325	7.306
PCO_2_ (mmHg)	19.2	51.1	58.5	65.5
PO_2_ (mmHg)	154	92.1	142	130
Beecf (mmol/L)	−0.3	0.1	4.1	5.7
HCO_3_-(mmol/L)	25.8	23.9	27.1	28.3
Na^+^ (mmol/L)	151	154	160	165
K^+^ (mmol/L)	3.4	4.0	3.9	4.0
iCa (mmol/L)	1.37	1.35	1.28	1.35
Glu (mmol/L)	5.4	7.1	6.9	6.3
Hb (g/dl)	12.2	11.6	11.4	11.6

Beecf, base excess of extracellular fluid; Glu, glucose; Hb, hemoglobin; HCO_3_-, bicarbonate ion; iCa, ionized calcium; K+, potassium ion; Na+, sodium ion; PCO_2_, partial pressure of carbon dioxide; PO_2_, partial pressure of oxygen.

## Discussion

ROHHAD was initially identified by Fishman et al. (5) and later renamed by Ize-Ludlow et al. in 2007 (6). The cause of this disease is currently unknown, and specific diagnostic tests are lacking, leading to a diagnosis that primarily relies on clinical manifestations (7).

The typical age of onset of this disease ranges from 2 to 7 years, with a peak incidence occurring between 2 and 4 years. The initial presentation often involves rapid weight gain. After a few months to years, children may develop hypothalamic dysfunction, leading to conditions such as growth hormone deficiency, inadequate water intake, hyperprolactinemia, hypothyroidism, and sodium metabolism disorders. Autonomic dysfunction may present with ophthalmic abnormalities, bradycardia, and excessive sweating. Behavioral changes include emotional fluctuations and attention-deficit hyperactivity disorder. Additionally, neurological abnormalities such as seizures and sleep disorders may occur, with seizures potentially linked to hypoxemia. Approximately 40%–56% of affected children develop neuroectodermal tumors such as neuroblastoma or ganglioneuroblastoma.

Respiratory insufficiency is a critical symptom of this disease, with many children experiencing obstructive sleep apnea, hypoxemia, and hypercapnia in early childhood. In severe cases, respiratory insufficiency may manifest when the child is awake. Owing to respiratory disorders and cardiopulmonary failure, the mortality rate of this disease is as high as 50%–60%. Treatment includes the provision of assisted ventilatory support, such as noninvasive positive pressure ventilation (mask ventilation) during sleep or positive pressure ventilation via tracheostomy when hypoventilation develops, and multidisciplinary care for various disease manifestations.

In this case, we have three important points of concern. At first, owing to this patient's preexisting ventilatory insufficiency, a 100% oxygen mask was administered during the induction phase. To minimize the duration of one-lung ventilation and prevent hypoxia, one-lung ventilation was initiated following the placement of the bronchial blocker prior to the thoracoscopic procedure. Reports suggest that the use of non-opioid analgesics and regional anesthesia may help prevent postoperative ventilatory insufficiency during extubation at the conclusion of surgery. Moreover, volatile anesthetics are recommended to expedite recovery and reduce the likelihood of postoperative airway obstruction (8). Thus during anesthetic induction, a low dose of 1 μg/kg of fentanyl was administered to minimize opioid usage. Sevoflurane was selected as the inhalational anesthetic to prevent respiratory depression and facilitate early extubation. The patient was transferred to the ICU for additional respiratory support following surgery. After extubation in the ICU, serial arterial blood gas tests performed on postoperative days 1 and 2 revealed respiratory acidosis with progressive hypercapnia, we recommend that this patient receive noninvasive positive pressure ventilation during sleep after discharge.

Secondly, the primary concern during surgery in patients with hypothalamic dysfunction is a significant decrease in thirst center function, which leads to hypernatremia. In this case, the patient underwent active hypotonic fluid therapy before surgery, with a sodium level of 155 mmol/L upon admission. During the surgical procedure, hypotonic fluid should be administered for sodium reduction therapy; however, the rate should be carefully controlled to prevent a rapid decrease in blood sodium levels, typically not exceed 0.5 mmol/(L·h), which could lead to cerebral edema. Intraoperative blood gas analysis revealed sodium levels fluctuating between 149 and 155 mmol/L, consistent with the preoperative levels. Furthermore, blood gas analysis showed elevated blood glucose levels, prompting the administration of a polarizing solution consisting of 500 ml of 5% glucose solution, 6 U of insulin, and 0.6 g of KCl. Arterial blood gas levels were closely monitored, urine output was assessed, and intraoperative fluid infusion was adjusted accordingly.

In addition, the patient displayed mild anemia before surgery, which may be attributed to the decreased red blood cell production caused by this syndrome. The large size of a tumor and its rich blood supply can result in intraoperative blood loss. Consequently, adequate blood products such as red blood cells and plasma were requested in advance. Following anesthesia, appropriate venous access was established to facilitate the administration of blood products and vasoactive drugs during surgery. Arterial monitoring was also initiated because of the potential for arrhythmias and autonomic dysfunction in these patients, which can lead to hypotension and hypertension during surgery. Invasive blood pressure monitoring was conducted to promptly detect changes in blood pressure, and arterial blood gas testing was performed.

This syndrome may manifest as anesthesia-related symptoms, including decreased gastrointestinal function, polyuria, abnormal temperature regulation, and diabetes in some affected children. While these symptoms were not evident in the current case, it is crucial to carefully consider them during anesthesia evaluation and implement necessary interventions, such as prolonging fasting duration, managing polyuria, monitoring body temperature, and regulating blood sugar levels.

In conclusion, the current lack of comprehensive reports on the anesthetic management of ROHHAD syndrome, coupled with the diverse preoperative presentations of affected children, underscores the importance of anesthesiologists in deepening their understanding of this condition. Tailored management strategies should be implemented based on distinct clinical manifestations and surgical procedures required for each child.

## Data Availability

The original contributions presented in the study are included in the article/Supplementary Material, further inquiries can be directed to the corresponding author.
